# The dynamic process of covalent and non-covalent PARylation in the maintenance of genome integrity: a focus on PARP inhibitors

**DOI:** 10.1093/narcan/zcad043

**Published:** 2023-08-21

**Authors:** Adèle Beneyton, Louis Nonfoux, Jean-Philippe Gagné, Amélie Rodrigue, Charu Kothari, Nurgul Atalay, Michael J Hendzel, Guy G Poirier, Jean-Yves Masson

**Affiliations:** CHU de Québec Research Center, HDQ Pavilion, Oncology Division, Laval University Cancer Research Center, 9 McMahon, Québec City, QC G1R 3S3, Canada; CHU de Québec Research Center, HDQ Pavilion, Oncology Division, Laval University Cancer Research Center, 9 McMahon, Québec City, QC G1R 3S3, Canada; CHU de Québec Research Center, CHUL Pavilion, Oncology Division, Laval University Cancer Research Center, 2705 Boulevard Laurier, Québec City, QC G1V 4G2, Canada; CHU de Québec Research Center, CHUL Pavilion, Oncology Division, Laval University Cancer Research Center, 2705 Boulevard Laurier, Québec City, QC G1V 4G2, Canada; CHU de Québec Research Center, HDQ Pavilion, Oncology Division, Laval University Cancer Research Center, 9 McMahon, Québec City, QC G1R 3S3, Canada; CHU de Québec Research Center, CHUL Pavilion, Oncology Division, Laval University Cancer Research Center, 2705 Boulevard Laurier, Québec City, QC G1V 4G2, Canada; CHU de Québec Research Center, HDQ Pavilion, Oncology Division, Laval University Cancer Research Center, 9 McMahon, Québec City, QC G1R 3S3, Canada; CHU de Québec Research Center, CHUL Pavilion, Oncology Division, Laval University Cancer Research Center, 2705 Boulevard Laurier, Québec City, QC G1V 4G2, Canada; Department of Oncology, Faculty of Medicine and Dentistry, University of Alberta, 11560 University Avenue, Edmonton, AlbertaT6G 1Z2, Canada; CHU de Québec Research Center, CHUL Pavilion, Oncology Division, Laval University Cancer Research Center, 2705 Boulevard Laurier, Québec City, QC G1V 4G2, Canada; CHU de Québec Research Center, HDQ Pavilion, Oncology Division, Laval University Cancer Research Center, 9 McMahon, Québec City, QC G1R 3S3, Canada

## Abstract

Poly(ADP-ribosylation) (PARylation) by poly(ADP-ribose) polymerases (PARPs) is a highly regulated process that consists of the covalent addition of polymers of ADP-ribose (PAR) through post-translational modifications of substrate proteins or non-covalent interactions with PAR via PAR binding domains and motifs, thereby reprogramming their functions. This modification is particularly known for its central role in the maintenance of genomic stability. However, how genomic integrity is controlled by an intricate interplay of covalent PARylation and non-covalent PAR binding remains largely unknown. Of importance, PARylation has caught recent attention for providing a mechanistic basis of synthetic lethality involving PARP inhibitors (PARPi), most notably in homologous recombination (HR)-deficient breast and ovarian tumors. The molecular mechanisms responsible for the anti-cancer effect of PARPi are thought to implicate both catalytic inhibition and trapping of PARP enzymes on DNA. However, the relative contribution of each on tumor-specific cytotoxicity is still unclear. It is paramount to understand these PAR-dependent mechanisms, given that resistance to PARPi is a challenge in the clinic. Deciphering the complex interplay between covalent PARylation and non-covalent PAR binding and defining how PARP trapping and non-trapping events contribute to PARPi anti-tumour activity is essential for developing improved therapeutic strategies. With this perspective, we review the current understanding of PARylation biology in the context of the DNA damage response (DDR) and the mechanisms underlying PARPi activity and resistance.

## Graphical Abstract

**Graphical Abstract:**
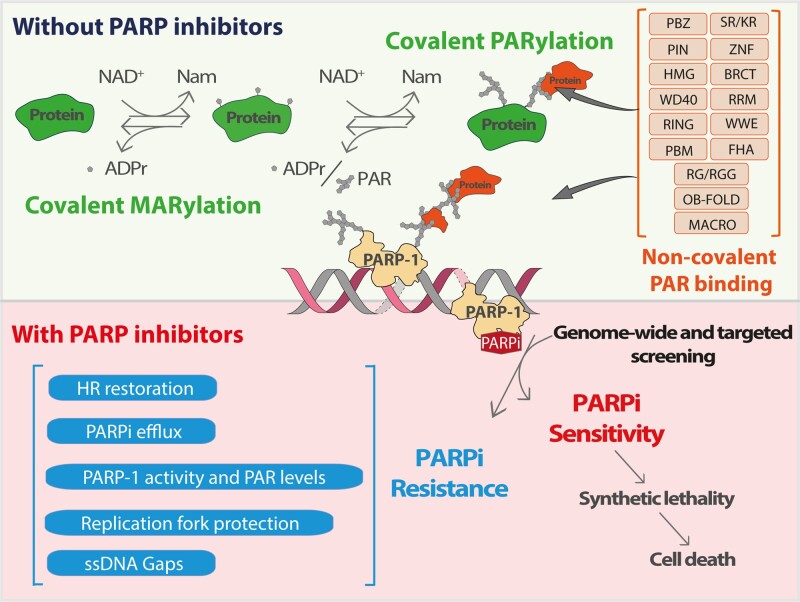

## INTRODUCTION

Poly(ADP-ribosyl)ation (PARylation) is a highly dynamic post-translational modification (PTM) that has gained increasing attention over recent years for its implication in many physiological and pathological processes and for emerging as a druggable target pathway for cancer therapy. It involves a series of transient and reversible attachment of polymers of ADP-ribose (PAR) covalently attached to various amino acid residues on protein substrates (1) thereby affecting their function, localization and stability (2). It is catalyzed by a subset of ADP-ribosyltransferases (ARTs), originally known as poly(ADP-ribose) polymerases (PARPs) (3,4), and is reversed primarily through the action of PAR-degrading enzymes referred to as PAR erasers (5,6). Poly(ADP-ribose) glycohydrolase (PARG) and the ADP-ribosylhydrolase ARH3 account for most of PAR degradation in cells and complement each other to regulate the dynamics of ADP-ribosylation (7). Several proteins also interact non-covalently with PAR through transient physical interactions to dictate many important biological events. In this view, PAR can be conceptualized as a molecular hub for recruiting various proteins including DNA repair factors, ubiquitin-conjugating enzymes and chromatin remodelers (8–11). Proteomic studies have identified thousands of covalently PARylated substrates and PAR binding proteins, adding extra layers of complexity to the PARylation process (12–35). Thanks to several decades of studies, covalent PARylation and non-covalent interactions with PAR are now recognized to play crucial regulatory roles in an impressive number of processes, including DNA damage response (DDR), DNA replication, maintenance of genome stability, modulation of chromatin architecture, and pathways related to inflammation, metabolism, protein degradation and cell death to mention a few (36). However, far less is known about how covalent PARylation and non-covalent PAR interactions influence each other to regulate these processes. The main knowledge about PARylation comes from the study of PARP-1, the prototype member of the PARP family (37,38), which has been best characterized for its role in the DDR and most particularly in DNA repair (39–42). In this context, PARP-1 is often described as a first responder that senses DNA breaks such as single- and double-strand DNA breaks (SSBs and DSBs) (43–45). It has been shown to initiate and modulate several DNA repair pathways, including nucleotide and base excision repair (NER/BER), both classical and alternative non-homologous end joining (C-NHEJ/A-NHEJ), homologous recombination (HR) and DNA mismatch repair (MMR) (46,47). Other DDR functions of PARP-1 include stabilizing replication forks and organizing chromatin structure (47).

Notwithstanding the importance of PARP-1 in numerous pathways that govern genome integrity, the effective killing of HR-deficient BRCA1/2-mutated cancer cells by PARP inhibitors (PARPi) has been the basis for the development of therapies targeting breast and ovarian tumors (48,49). Trapping PARP-1 on specific DNA lesions, including repair intermediates, is the prevailing model explaining how PARPi effectively kills HR-defective cells (50). PARPi exploits tumour-specific defects in HR repair through the concept of synthetic lethality (51). However, there is now clinical evidence to support their use in other molecular subsets of cancers beyond HR-deficient BRCA1/2-mutant cancers (52,53) or in combination with other targeted drugs to potentiate the clinical efficacy of modern cancer therapies (54–56). Despite the FDA-approval of PARPi to treat a variety of cancers (e.g. ovarian, breast, prostate or pancreatic cancers), resistance to PARPi has proved to be a major challenge. More than 40% of BRCA1/2-mutated ovarian cancers treated in the clinic fail to respond to PARPi (57). Some patients with confirmed BRCA1/2 mutations respond poorly to PARPi while others, with no apparent BRCA defect, respond well to PARPi therapy (58). Variable patient responses highlight the inadequate understanding of synthetic lethal interactions. A clear picture of the molecular mechanisms underlying PARPi is necessary to assess the full benefit of PARPi in cancer therapy.

This review discusses new advances in our understanding of covalent PARylation and non-covalent PAR binding in the context of the DDR and how the dynamic interplay between these effects can contribute to PARPi-based therapies. We also highlight the current evidence connecting PARylation events to PARPi patient response.

## STRUCTURE–FUNCTION RELATIONSHIPS OF PARPS

PARPs, also referred to as diphtheria-toxin-like ARTs (ARTDs) (3,4), share a conserved catalytic domain that enables the binding of nicotinamide adenine dinucleotide (NAD^+^) and transfer of ADP-ribose moieties from donor NAD^+^ to protein acceptors (1,59). While a majority of ARTs are restricted to the covalent attachment of a single mono(ADP-ribose) moiety (MAR) to a target amino acid residue (MARylation), others possess protein-distal ADP-ribose polymerization (i.e. chain elongation) activity that allows further addition of ADP-ribose units through ribose–ribose glycosidic bonds, creating negatively charged PAR chains of variable length and branching frequency (PARylation) (38). However, ARTs tend to defy rigid classification as to whether they are MARylating or PARylating enzymes since the distinction between the two sub-families has mostly been based on their structural features and auto-ADP-ribosylation activity (38,60). So far, among the 17-member PARP family, only PARP-1, PARP-2, Tankyrase-1 (TNKS-1, PARP5a) and Tankyrase-2 (TNKS-2, PARP5b) have unambiguously been identified to synthesize PAR chains (i.e. considered to be *bona fide* PAR writers), while the other members are restricted to MARylation activity or are catalytically inactive (38,61). Besides, a family-wide analysis of ARTs activity indicated that the highly conserved H-Y-E motif found throughout the catalytic (CAT) domain of the ARTDs superfamily is not the sole indicator of PARP activity (38). *In vitro* studies have shown that PARP-1 and PARP-2 synthesize long branched chains (as many as 200 units with branching occurring every 20–50 ADP-units in the case of PARP-1), whereas TNKS-1 and TNKS-2 produce shorter chains of up to 20 units with no detectable branching (11,62,63). Notably, PARP-2 possesses a higher branching rate than PARP-1 and promotes branched PAR formation by PARP-1 (63). The biological significance of the substantial chain length and branching frequency heterogeneity frequencies of PAR is largely unknown. However, a recent study using a short hypobranched PARP-1 mutant suggested a role for PAR chain length and branching in cellular physiology and stress response (64). PARP-1 is the most abundant and active PARP enzyme, accounting for approximately 90% of the total PAR synthesis in response to DNA damage, and also a main target of PARylation through automodification (65,66). PARP-1 is found ubiquitously in the nucleus where it binds to and is catalytically activated by SSBs and DSBs, as well as other DNA alterations and structures (67). In addition to PTMs, various cofactors and effectors were shown to modulate the level and specificity of the PARylation activity in cells (25,68–70) . Once activated, PARPs PARylate several target molecules including DNA (71–73), RNA (74–76) and key DDR proteins such as DNA repair and chromatin regulatory proteins, a phenomenon referred to as PAR spraying (77). The high-density deposition of PAR generates a transient repair compartment that concentrates repair proteins and activates signaling factors (78,79). PARP-1 automodification also causes its release from DNA (80) and promotes chromatin relaxation, a crucial process for downstream repair events (81–83).

**Figure 1.**
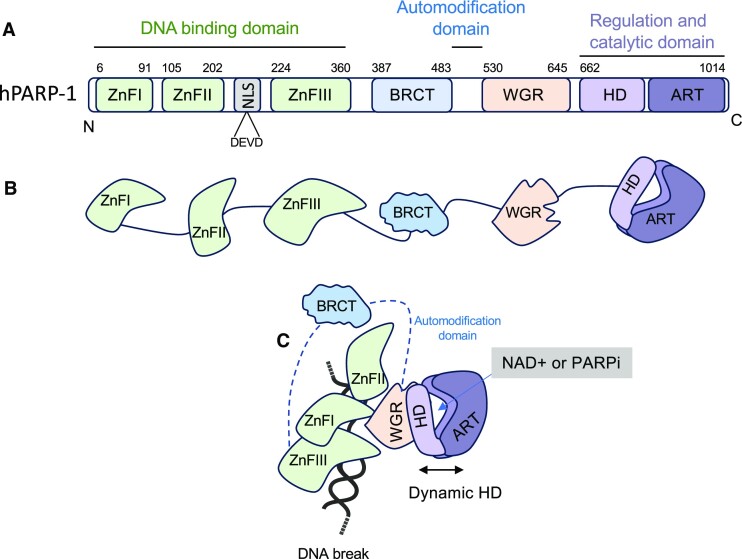
(**A**) Human PARP-1 protein domains. PARP-1 bears six independently folded domains connected by flexible linker regions. The nucleic acid-binding region contains three zinc finger domains (ZnFI, ZnFII, ZnFIII, in green); a BRCA C-terminus (BRCT)-containing automodification domain (in blue); a nucleic acid-binding motif tryptophan– glycine–arginine (WGR, in orange) and the catalytic domain at the C-terminus, composed autoinhibitory helical domain (HD, light purple) and the ADP-ribosyl transferase fold (ART, dark purple), allowing PARP-1 to convert NAD+ into poly(ADP-ribose). (**B**) Communication between domains is essential for its DNA damage-dependent catalytic activity (modified from reference (334)). PARP-1 binding to DNA damage leads to systematic domain rearrangements, and allosterically leads to a dynamic HD (shown by double arrows). The binding to NAD+ (or PARPi) requires an open HD conformation leading to increased interaction with DNA.

Structurally, human PARP-1 is a 1014 amino acids protein of 113-kDa consisting of three zinc fingers (ZnFI, ZnFII and ZnFIII), a BRCA1 C-Terminus (BRCT) domain, a Trp-Gly-Arg (WGR) domain, a C-terminus catalytic domain (CAT) composed of an alpha-helical subdomain (HD) and an ADP-ribosyl transferase subdomain (ART). Also found at the N-terminus is a nuclear localization signal (NLS) region containing the caspase cleavage site ^211^DEVD^214^, which is cleaved during apoptosis (84) (Figure 1A). ZnFI and ZnFII are required for recognition and binding to DNA damage sites. Two different arguments circulate regarding the role of ZnFII in DNA binding. The first argument states that ZnFI and ZnFII bind DNA as dimers (85,86). In support of this, mutational and deletion analyses of the ZnFI and ZnFII domains have indicated that both domains possess DNA-binding activity, with ZnFI being also essential for PARP-1 DNA-dependent activity (86,87). ZnFI residue D45, which does not have an equivalent in ZnFII, is critical for PARylation, providing a molecular basis for the specificity of ZnFI in regulating PARP-1 activity (87). Ali *et al.* have proposed a model for the cooperative binding of ZnFI and ZnFII to DSBs, where ZnFI contacts DNA via R18 (in the phosphate backbone grip) and F44 (at the base stacking loop), whereas ZnFII binds with R122 (in the phosphate backbone grip) and L161/I164 (at the base stacking loop) (86). On the contrary, some groups have argued that PARP-1 binds to DNA as a monomer (88,89). Nevertheless, these studies have collectively suggested that ZnFI is indispensable for the activity of PARP-1, whereas ZnFI can compensate for ZnFII activity. The third ZnF domain (90), ZnFIII, mediates the DNA-dependent PARP-1 activity by interacting with ZnFI on one side and the WGR domain on the other. Deletion of ZnFIII does not affect the DNA binding capacity of PARP-1 but substantially decreases its enzymatic activity. More specifically, amino acids W318 and T316 of ZnFIII are critical for the enzymatic activity of PARP-1 (91).

Formerly described as the automodification domain, the BRCT domain regulates protein–protein interactions and was recently found to bind to intact DNA without activation of PARP-1 and to mediate rapid movement of PARP-1 through the nucleus (92). There is now a body of evidence that suggests that the predominant automodification site of PARP-1 is located in a flexible interdomain loop that connects the BRCT to the WRG domain (15,30,32,93,94). The WGR domain, along with ZnFI and ZnFIII, interacts with DNA, bridging the DNA damage interface and the CAT domain, which is important for PARP-1 activity (88). The CAT domain is responsible for NAD^+^ hydrolysis, the attachment of the first ADP-ribose to an acceptor amino acid residue, followed by elongation and branching of the PAR (95). Finally, the HD domain acts as an inhibitor of the ART domain (96). Destabilization of the HD enables the activation of the ART subdomain. It has been shown that deletion of the HD domain constitutively activates PARP-1, even in the absence of DNA (96). Following binding to DNA damage, PARP-1 undergoes systematic domain rearrangements, which allosterically leads to a dynamic HD. The binding to NAD+ (or PARPi) requires an open HD conformation, leading to increased interaction with DNA (Figure 1B). The ART domain embodies a well-conserved sequence called the PARP signature motif (37,97), which carries the NAD^+^ binding site and other conserved catalytic structures required for PAR initiation, elongation and branching.

Like PARP-1, activation of PARP-2 and PARP-3 is also DNA-dependent (80). Following PARP-1, PARP-2 is the next major contributor to PAR synthesis and can partially compensate for PARP-1 activity. Human PARP-2 (583 amino acids) is relatively shorter than PARP-1 and only possesses a very short N-terminal extension in addition to WGR and CAT domains with high similarity to PARP-1. The N-terminal domain of PARP-2 mediates interaction with DNA but cooperates with both the WGR and CAT to maximize the binding affinity (98,99). The resulting destabilization of the HD domain activates the CAT domain and triggers its PARylation activity. Besides the structural similarity and the interchangeable roles of PARP-1 and PARP-2 in DNA damage, these PARP family members also have important and non-overlapping functions. A wide variety of DNA structures have been identified as PARP-1 activating substrates (e.g. single- and double-strand breaks, unligated Okazaki fragments, overhangs, hairpins, cruciforms, etc.), while PARP-2 is activated by a more restricted spectrum of nucleic acids (e.g. 5'-phosphorylated single-strand DNA or single-stranded RNA (80,100)). PARP-1 and PARP-2 can bind a variety of RNAs but there is still some controversy regarding the question of whether this can lead to catalytic activation (101). The affinity of PARP-1 for RNA has been sparsely reported in recent decades (102–104). In the last 10 years, there has been a new enthusiasm for the study of PARylation-mediated events to control mRNA processing (105–107), RNA biogenesis (108–110) and DNA/RNA hybrid resolution (111). Small nucleolar RNAs (snoRNAs) were also reported as important DNA damage-independent activators of PARP-1 activity in the control of ribosome biogenesis (112), a pathway that may contribute to the effectiveness of PARPi in the treatment of a number of cancer types (113,114). Finally, it should be noted that, in contrast to PARP-1, PAR polymers can robustly activate PARP-2 activity, which suggests a tight connection between both activities in the DDR (63). Clearly, the control of PARP-1 activation involves a complex orchestration of several types of nucleic acid interactions.

PARylation heterogeneity arises from variable polymers structures (i.e. length and branching frequencies), the nature of the ADP-ribose linkages (i.e. the acceptor amino acid residue) and different degrees of occupancy (i.e. the molecular stoichiometry of the protein–PARylation reaction or the number of ADP-ribose chains/polypeptide unit) (115). However, the underlying determinants that modulate PARP-1 activity and PAR complexity in various physiological contexts remain largely unexplored. A recent study by Krüger *et al.* shed some light on this phenomenon by using attenuated total reflectance-Fourier transform infrared spectroscopy (ATR-FTIR) to demonstrate the binding affinity and activation potential of PARP-1 at different DNA strand breaks in a real-time fashion (67). The study demonstrated that, although the binding and activation of PARP-1 showed similar kinetics at different DNA strand break models, there were significant differences in the PARP-1 PARylation reaction and dissociation processes from the different structures. The strongest PAR formation with systematic dissociation was observed at nicks and 3'-phosphorylated DNA ends, while a weaker activation was observed at 5'-phosphorylated ends. Krüger and colleagues also showed that besides the WGR, NAD^+^ binding also destabilizes the HD, explaining the faster rate of PAR elongation compared to PAR initiation. Decoding the combinatorial PARylation pattern involving PAR polymers of varying attachment sites, lengths and structures is a major obstacle to understanding PAR-regulated pathways. The interplay between PAR writers, readers and erasers creates potential for tremendous PAR heterogeneity and limits our ability to understand how it contributes biologically to complex systems.

## COVALENT PARYLATION AND NON-COVALENT PAR binding

PARylation is unique among PTMs because molecular interactions with PAR are likely as important as covalent PARylation in impacting protein functions. Similar to other complex biopolymers, such as glycans, the physical attachment of the polymer to a protein is not the sole way to manipulate its biological activity (116). However, in contrast to saccharide polymers, a much wider range of proteins have evolved to bind PAR in a manner specific to their function. In this section, we describe the molecular events associated with each process and how the crosstalk effect between them contributes to the PARylation of a plethora of substrates involved in a variety of cellular pathways.

### Covalent PARylation

The link between PARylation and chromatin followed soon after the discovery of this biopolymer in the nucleus of cells (117–119). Although there was some confusion at the time about whether nuclear PAR was free or protein-bound, subsequent studies rapidly showed that histones and nuclear proteins were covalently PARylated (120–126). The release of protein-free PAR fragments by the endoglycosidic activity of PARG (127–129) was described later as a PAR-dependent cell death pathway (also known as parthanatos) (130,131). Studies on purified nucleosomes suggested that PARP-1 was preferentially localized into internucleosomal regions of the nucleosomes (132,133). This observation was consistent with the identification of the linker histone H1 as a major PARylated PARP-1 substrate (134–136). Histone H1 hyper-PARylation is responsible for rapid polynucleosome relaxation (137,138). Although initial studies suggested that no additional histones apart from H1 were major acceptors of PAR, subsequent studies revealed that all core histones were also PARylated, albeit more modestly (139,140).

**Figure 2.**
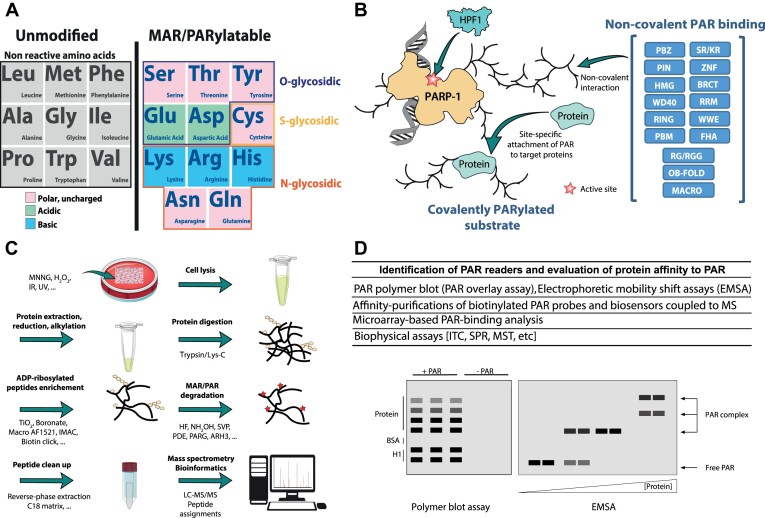
(**A**) ADP-ribose can be covalently attached to various amino acid residues. Unmodified versus MAR/PARylatable residues are shown. Polar uncharged amino acids are in pink; acidic amino acids are in green; and basic amino acids are in blue. The type of glycosidic bonds is also shown. (**B**) Diagram showing the activation of PARP by HPF1 and the formation of poly(ADP-ribose) which is bound non-covalently by PAR binding domains. The covalent addition of poly(ADP-ribose) on proteins is also shown. (**C**) Assessment of PARylated residues following DNA damage by mass spectrometry. (**D**) Techniques used to detect PAR readers and evaluate protein affinity to PAR.

Following the pioneering work of Adamietz and Hilz, ADP-ribose-protein linkages were classified into two sub-categories based on their sensitivity to hydroxylamine hydrolysis (141). The carboxyl-ester type of bonds linking ADP-ribose to glutamate (GLU) and aspartate residues (ASP), each having a carboxylic acid on its side chain, are unstable when exposed to hydroxylamine and were thus characterized as hydroxylamine-labile. On the other hand, the ketamine linkages established between the ADP-ribose and the amine group of lysine (LYS) (142), or the guanidino group of arginine (ARG) (143), were designated as hydroxylamine-resistant. In addition to the Adamietz and Hilz classification, other types of bonds, also hydroxylamine-resistant, have been reported when ADP-ribose is attached to a cysteine residue (CYS) by a thioglycosidic bond (38,144) or to serine (SER) (145), threonine (THR) or tyrosine (TYR) (25,146) via O-glycosidic acetal linkages (147,148). Alanine, glycine, isoleucine, leucine, methionine, phenylalanine, proline, tryptophan, and valine are not reactive to MARylation or PARylation (Figure 2A). At the time, the vast majority of studies focusing on the nature of the ADP-ribose linkages provided evidence that ADP-ribosylated histones and PARP-1 itself were primarily modified via hydroxylamine-sensitive carboxyl-ester type of bonds (149–154). Although ADP-ribosylation was mainly associated with ASP/GLU residues, most of these studies have not directly identified the specific modification sites. However, the more recent identification of a cluster of ASP/GLU residues at the nucleosomal surface as the main target of histone ADP-ribosylation and the observation that GLU/ASP-ADP-ribosylation sites on histones are mutated in cancers suggest an important functional link between GLU/ASP-ADP-ribosylation and cancer (77,155,156).

The identification of HPF1 (Histone PARylation factor 1) as an important regulator of PARP-1 activity changed our vision of PARylation (94,157–159). HPF1 remodels the catalytic site of PARP-1 and switches the nature of the ADP-ribose linkages it generates (160). Although the exact context in which HPF1 reshapes the catalytic site of PARP-1 in response to DNA damage is still poorly understood, it switches PARP-1 ADP-ribosylation from carboxylate esters linkages (ASP/GLU residues) to acetal linkages (SER, THR, TYR) (161). Accumulating evidence suggests that acetal PARylation coordinates DNA repair with cell cycle progression to maintain genome stability (162–169). Notably, HPF1-dependent histone ADP-ribosylation by PARP-1 contributes to DNA damage-induced chromatin relaxation and promotes the recruitment of repair factors at sites of DNA damage (163). On the other hand, carboxylate ester linkages formed between ASP/GLU residues are also closely linked to the DDR (15,28). The co-occurrence of both types of ADP-ribose linkages on PARylated substrates is still debated. It remains unclear whether these PTMs are cooperative, mutually exclusive, spatio-temporaly restricted or if they can influence or block the addition of another ADP-ribosylation at a nearby site. We hypothesize that linkage-specific ADP-ribosylation profiles will likely orchestrate the DDR (62,64). Because an interplay exists between SER-ADP-ribosylation and histone marks (146), it is reasonable to think similar dynamics apply to ASP/GLU-ADP-ribosylation (155,170).

Similar to many other PTMs, PARylation requires enrichment prior to mass spectrometry (MS) analysis because of a relatively low stoichiometry in cells. As a consequence of the intrinsic molecular heterogeneity of PAR, several ADP-ribosylation derivatization strategies were developed to generate mass-specific ADP-ribose signatures for MS analysis. A specific challenge to ADP-ribosylation analysis is that MS dissociation methods must fragment the ADP-ribosylated peptide backbone while preserving the labile modification from being lost (17,147,171). In 2013, the research group led by Yonghao Yu generated the first large-scale study of site-specific ADP-ribosylation sites (15). The method combined boronate affinity-purification with hydroxylamine hydrolysis to convert ASP/GLU-ADP-ribosylation into a unique hydroxamic acid spectral signature. The major advantage of this method resides in its straightforward applicability to MS analysis because the labile ADP-ribose group is converted to a small hydroxamic acid remnant that does not interfere with peptide backbone fragmentation using collision-induced dissociation (CID) which is a common and robust fragmentation method used in MS. However, the method is limited to hydroxylamine-sensitive ADP-ribosylation and cannot provide MS/MS filtering possibilities based on the presence of diagnostic ions generated by the fragmentation of the intact ADP-ribose. As an alternative approach to identify site-specific ADP-ribosylation sites by MS, other groups used hybrid fragmentation techniques that combined higher-energy collisional dissociation (HCD) and electron-transfer dissociation (ETD) (26,27,31,172), a fragmentation scheme successfully applied to proteome-wide analysis of protein glycosylation (173). The method allowed to access both the ADP-ribose and peptide information on targeted residues, irrespective of their linkage specificity. Globally, large-scale proteome analysis of ADP-ribosylation based on this approach revealed that a large fraction of the nuclear proteome is modified by SER-linked ADP-ribosylation (27,31,32,174). A database of ADP-ribosylated proteins maintained by the group of Anthony Leung currently houses over 9000 PARylated proteins (175,176).

### Non-covalent PAR binding

PARylation is a complex process assisted by different writers, readers and erasers to generate a wide variety of structural variations. The biological role of specific PAR structures requires further exploration but the growing identification of protein motifs, domains and modules that exhibit high affinity to PAR is consistent with the structural diversity of PAR in biological systems (62).

Intermolecular interactions between PAR and protein modules can be established via non-covalent binding. Several PAR readers interact with PAR with affinities in the nanomolar range (35,177–179). This was first demonstrated for nuclear proteins by the landmark paper of Panzeter and colleagues showing that histones and protamines bind branched PAR polymers with greater affinity than linear polymers (180). Remarkably, histone–PAR complexes were resistant to strong acids, chaotropes, detergents, high salts concentrations and phenol-partitioning, but the interactions were reversible with DNA competition. The high affinity between histones and PAR contributes to the transient chromatin relaxation and histone displacement observed upon DNA damage. This concept was further extended to non-histone proteins such as p53, DNA-PK or KU70/80 and led to the definition of a common polymer-binding motif of 22–26 amino acids that conveyed the specific affinity for PAR. This motif contains a cluster of positively-charged residues in a consensus pattern [hxbxhhbbhhb] where h indicates residues with hydrophobic side chains, b stands for a preference for basic amino acids, and x for any amino acids (181). The motif was further refined to a sequence with a restricted set of amino acids at the conserved site formulating a new sequence as [HKR]-X-X-[AIQVY]-[KR]-[KR]-[AILV]-[FILPV] (12,182) where the two positively charged amino acids residues [KR]-[KR] are strictly followed by either A, I, L or V ([AILV]) which are classified as residues with alkyl side chains. The consensus PAR binding motifs (PBMs) are derived from *in vitro* experiments and demonstrated to mediate protein PAR binding in cells (12,182). However, motif refinements are still required for improved binding sites predictions. PBMs and other PAR-reading modules are not limited to the nuclear compartment as numerous nonchromatin proteins can bear one or more domains that recognize PAR. A notable example is the inhibition of the cytoplasmic and mitochondrial protein hexokinase 1 (HK1) upon non-covalent PAR binding, an interaction that blocks glycolysis, which culminates in a cellular bioenergetic collapse (183,184).

A growing body of research now indicates that PAR binding modules are found in a wide variety of proteins and in a variety of different arrangements with other functional domains (128,185,186). For example, a subset of C_2_H_2_-type zinc finger domains can be specifically involved in PAR recognition as it has been demonstrated for CTCF (187). This type of zinc finger domain is extremely common in the human proteome and found as repeats in a plethora of proteins engaged in transactions with nucleic acids. It is important to realize that multiple types of interactions are possible and might affect the equilibrium constant and contribute to the apparent affinity to PAR. A PAR reader may have one or more binding sites for specific ADP-ribose structures. PAR readers can also exhibit multivalent interactions with PAR ligands. PAR valency, which refers to the number of ADP-ribose binding sites per molecule, is increased by PAR length and branching frequency. This concept predicts that the affinity of a PAR reader can be increased by multivalent binding and domain cooperation, as it is mostly likely the case for lysine-rich histones and zinc finger proteins, respectively.

Most PAR reading modules are multifunctional domains that are not only capable of recognizing and binding to PAR but also mediating interaction with other biomolecules. This is obviously the case with zinc finger proteins, including the RING finger domain. However, this definition can be generalized to several other PAR binding domains such as the BRCT domain (protein-protein interaction) (188,189), RNA recognition modules (RRMs) (182,190), the WD40 domain (191), the glycine-arginine-rich (GAR) domains (RNA binding) (43,182), the forkhead-associated (FHA) domain (phosphopeptide binding) (188), the oligonucleotide/oligosaccharide-binding fold (OB-fold) (ssDNA or RNA binding) (192), or the WWE domains (protein-protein interactions) (193). Other modules seem to be more specifically involved in MAR/PAR recognition, such as the PAR binding zinc finger (PBZ) module (194), the macro domains (177) or the PIN (PAR-interacting) domain (195) (Figure 2B). Some of these binding motifs have nanomolar affinity constants with very low dissociation rates, highlighting their use as PAR biosensors to detect PARylation events in live cells (62,196,197).

Forming a complex matrix of PAR binding proteins and PAR polymers may play a crucial role in regulating the DDR. Specialized PAR readers, such as those containing the aforementioned PAR recognition modules, might contribute to the PAR-seeded liquid–liquid phase separation (LLPS), a demixing process that concentrates DDR factors and DNA repair proteins to orchestrate the DNA repair machinery at the lesion site (198–202). Intrinsically disordered proteins accumulate at sites of DNA damage in a PAR-dependent manner to generate LLPS. Considering that one-third of all eukaryotic proteins have been reported to contain at least one functionally relevant long intrinsically disordered region (203), one can imagine the levels of complexity reached in PAR-associated protein networks. With this many covalently PARylated and PAR-reading players, conventional methods of detecting specific protein interactions with PAR need to be improved and adapted to the context of PARylation.

### Crosstalk between covalent PARylation and non-covalent PAR binding

Covalent and non-covalent PARylation have a high level of co-occurrence in proteins. This is particularly true for proteins targeted to the local DNA damage site where PAR spraying coordinates the DDR (77). The synthesis of a large mesh of PAR at DNA lesions, due to the activation of DNA-dependent PARPs, has been conceived as a loading platform for various DNA damage response factors and an essential scaffold to recruit components of the DNA repair machinery. PAR is the most electronegative natural polymer. Local clustering of PAR, which harbors twice the negative charge density of single-stranded DNA (204), plays a significant role in determining the extent of chromatin decompaction at DNA lesions. By virtue of their long-chain nature, the negatively charged polymers of ADP-ribose (polyanions) are particularly prone to cause steric hindrance for anionic interaction with chromatin. ADP-ribosylation alters the electrostatic charge density of lysine-rich histone proteins, a factor that weakens histone-DNA interactions and promote chromatin remodeling. Moreover, apart from its chromatin de-packaging functions *per se*, ADP-ribose polymers also acts as adaptors to recruit factors that are directly involved in chromatin remodeling and DNA repair. Of note, complex PAR polymers can be larger than the protein substrate they are attached to and switch the protein to a less stable conformation.

PARP-1/2 can PARylate themselves covalently in *cis* (automodification) or *trans*-PARylate several sensors, transducers and effectors that orchestrates the DDR. In addition, PAR polymers can be bound by target proteins through high-affinity non-covalent interactions. Collectively referred to as PAR readers, these PAR binding proteins engage in a dynamic interplay with PAR writers and erasers to control the tightly orchestrated processing events that regulates PARylation in cells. In contrast to most PTMs, non-covalent binding to PAR proved to be as important as covalent PARylation to reprogram protein functions. A major challenge in PARylation studies is integrating and tracking both phenomena in a complex regulatory protein network. Often, both contingencies must be considered to understand PAR-regulated pathways.

The crosstalk between non-covalent PAR binding and covalent PARylation can be illustrated by the activity of the C-terminal domain (CTD) of p53 which orchestrates the interplay between both processes. In a series of experiments, Fischbach and colleagues showed that the CTD domain of p53 is a PAR binding module that is also essential for the covalent PARylation of p53 by PARP-1 (205). They also showed that fusing the CTD PAR recognition domain of p53 to a protein normally not PARylated renders this a target for covalent PARylation. The authors proposed a model in which covalently PARylated PARP-1 substrates are first attracted to the vicinity of automodified PARP-1 by their affinity to PAR and then brought near the catalytic site of PARP-1 by a sliding mechanism. A similar PAR-mediated interaction between PARP-1 and the RNA helicase DDX21 also leads to covalent ADP-Ribosylation of DDX21 (112). This model also implies that a rigid consensus sequence for selecting the covalent PARylation site would not be required, which could explain the PARylation of different amino acid acceptors. However, a certain preference for a proline-directed motif exists for GLU-ADP-Ribosylation ([PxE][EP][PxxE]) (15), while the large majority of SER-ADP-Ribosylation resides within [KS] motifs (32). Although the sliding mechanism cannot be assigned to all PAR recognition modules and PAR binding motifs because they engage with PAR through different structures and mechanisms, it helps explain the overlap between non-covalent PAR binding proteins and covalently PARylated substrates identified in several MS-based studies. The rapid turnover of PAR by erasers at DNA lesions could also facilitate multiple waves of DNA repair factor recruitment and additional PARP-1 accumulation at damage sites to covalently PARylate protein substrates in subsequent rounds of PARylation (6). A slower wave of PARP-1 activity that regulates DNA repair was identified by the group of Matic and supports this idea (206). The inability to find a unique model to describe the broad substrate diversity of covalently PARylated proteins is consistent with a multifactorial model that integrates several of the elements described above.

The non-covalent PAR binding events are of very high affinity and can persist during SDS-PAGE. As mentioned before, very strong and long-lasting interactions with PAR can resist to harsh denaturing conditions (180). Several approaches can be used to distinguish covalent versus non-covalent ADP-ribosylation but only tandem mass spectrometry (MS/MS) can provide decisive evidence of site-specific covalent ADP-ribosylation. (Figure 2C). Although PAR is significantly less flexible than DNA and RNA (207), the formation of PAR-protein complexes can be studied with virtually all the tools developed for nucleic acids since PAR shares many of their biophysical properties (208,209). Similar to DNA, filter binding and electrophoretic mobility shift assays (EMSA) were used to provide a rapid evaluation of the affinity of a protein for PAR (178,210). More quantitative and sensitive methods such as surface plasmon resonance (SPR) (178), isothermal titration calorimetry (ITC) (177) or spectroscopy techniques (211) were also carried out to evaluate PAR binding affinities. The polymer-blot assay (also known as filter-binding or PAR-overlay assay) is one of the most frequently used methods to characterize PAR binding proteins and evaluate their non-covalent affinity for PAR (212–214) (Figure 2D). Here, peptides encompassing PAR binding motifs or purified proteins are immobilized onto membranes either by manual spotting or transblot procedures. Protein–PAR interactions can be detected using a radio-labeled PAR probe or an anti-PAR antibody (similar to the far-Western blotting technique) (214). Protein microarray technology can also be used to investigate PAR and protein interactions on a proteome-wide scale.

MS has been instrumental in defining proteome-wide views of PARylation-dependent biological processes. The first approaches based on the isolation of PAR-containing multiprotein complexes in nondenaturing conditions were limited by the inability to discriminate between a covalently PARylated substrate, a noncovalent PAR binding factor or a secondary partner in a complex network of interacting proteins (12,13). Several MS-based methods were developed to assess the site-specific ADP-ribosylation proteome with increasing sensitivity and specificity, but identifying the PAR reading proteome has not been pursued as extensively. However, new methods are emerging to enable the specific identification of non-covalent PAR binding proteins, in a proteome-wide manner, by MS analysis. Recently, Dasovich and colleagues developed an elegant approach to investigate the non-covalent PAR proteome (35). The authors based their method on a photoaffinity probe, PARprolink, consisting of PAR chains of a predefined length, a biotin handle at the 2'-OH-terminus of PAR and a photo-crosslinker to stabilize PAR interactions. Crosslinked proteins are affinity-purified using streptavidin-coated beads and identified by mass spectrometry to generate a repertoire of non-covalent PAR binding proteins. ADP-ribose probes were also used to generate MAR and PAR interaction maps (33). Although the proteomic datasets associated with non-covalent interactions with PAR are currently less extensive than those aimed at identifying covalently PARylated substrates, there is still significant overlap between the two modes. A striking example is the PAR binding protein FUS whose strong affinity to PAR has been validated in several studies (215–217). FUS was also identified with hundreds of MS/MS spectra that bear site-specific ADP-ribosylation signatures (mostly on ASP and GLU residues) (15,28). The presence of intrinsically disordered regions in FUS, which were proposed to provide the ability of the substrate protein to process along PAR chains (205), supports the PAR sliding model and exemplifies the intricate relationship between covalent and non-covalent PARylation. However, the biological significance of such interrelation is still poorly understood. Very few site-directed mutagenesis studies identified specific amino acid positions that are important for PAR binding or those functionally important in the context of covalent PARylation.

## PARPI, PARP TRAPPING AND CANCER

**Figure 3.**
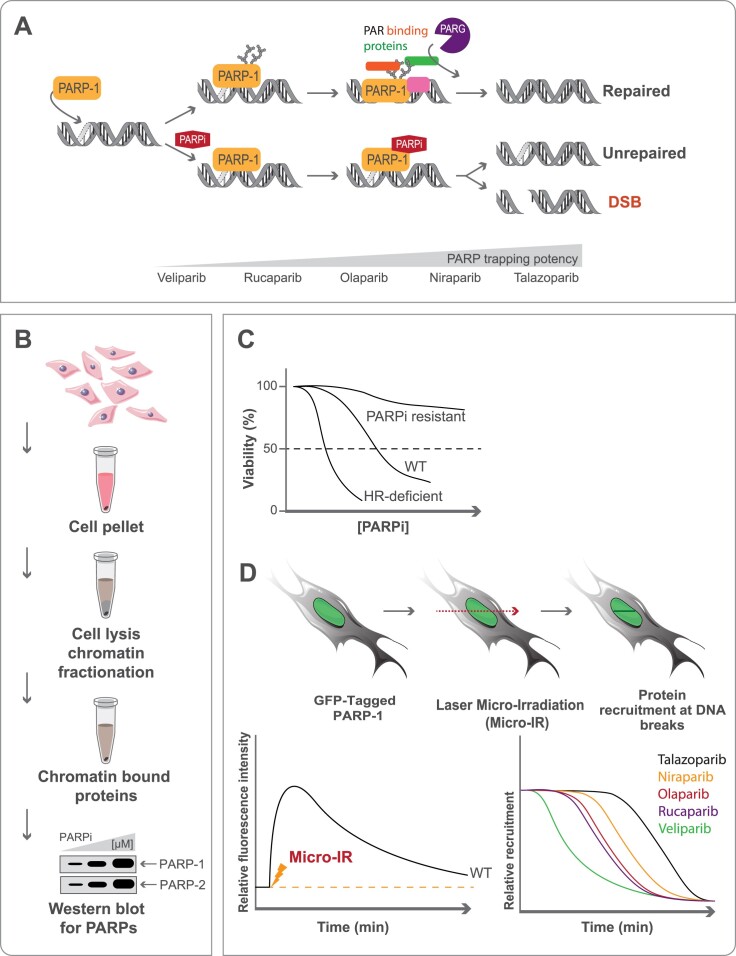
(**A**) PARPi have different potencies to trap PARP-1. PARPi trapping can lead to unrepaired DNA or DNA double-strand breaks. PARP trapping can be monitored by chromatin enrichment and western blotting (**B**); indirectly by cell viability assays (**C**) or by impaired mobility at DNA double-strand breaks following laser micro-irradiation (**D**). The dissociation of GFP-tagged PARP will be affected differently depending on PARPi trapping potency.

Several mechanisms have been suggested to explain the therapeutic effect of PARPi, each focusing on a specific aspect of the biology of PARP-1. One model, in particular, stood out since it could explain why BRCA1/2-defective cells are far more sensitive to PARPi than to the siRNA knockdown of PARP-1. In 2012, the group of Yves Pommier introduced the concept of PARP trapping, a poisoning model where PARP-1 becomes irreversibly bound to DNA damage sites (50). This model was developed around the idea that PARP-1 locked onto DNA would cause a dysfunction of the DNA repair machinery that ultimately leads to the accumulation of toxic DNA repair intermediates. The correlation between PARP trapping and tumor sensitivity provided a strong argument for the PARP-1 trapping model. PARPi have different potencies to trap PARP-1, talazoparib being the most potent and veliparib being the least active (218) (Figure 3A). Evidence obtained from recent research has challenged this theory. The current model is constantly evolving to introduce additional elements to the trapping mechanism (219–223). It now appears that the concept of PARP-1 trapping also involves a protein network and a multi-factorial dynamic that has been underestimated. For example, PARP-1 SUMOylation and ubiquitination in PARPi-induced trapping have been shown to dictate the efficacy of PARPi in cancer cells (224). Also, according to the poisoning model, cells or tissues with high PARP-1 expression should be hypersensitive to PARPi. Generally, the levels and patterns of PARylation correlate with PARPi sensitivity and clinical outcomes in ovarian cancers (225). However, some PARPi-resistant ovarian cancer cell lines with relatively high PARP-1 expression levels have low PAR levels, indicating that the cellular genetic background influences PARP-1 activity and PAR turnover. Finally, PARPi sensitivity is not always predicted by HR deficiency-related genomic signatures. The inconsistencies between the cellular response to PARPi observed in some tumors and the current trapping model indicate that more studies are required to understand the molecular mechanisms contributing to PARPi efficacy.

PARP trapping can be assessed by several orthogonal methods (220). Chromatin-bound fractions can be subjected to PARP-1 western blotting and quantification after the drug treatments (Figure 3B). Furthermore, PARP-1 trapping can also be monitored by live-cell microscopy using GFP-tagged PARP-1/2 and UV or near-infrared microirradiation in the absence or presence of PARPi (Figure 3C). The connection between PARP-1 trapping and tumour sensitivity is still under debate. While there is a correlation between the PARP-1 trapping activity of PARPi and their toxicity in cell lines, three different PARPi exhibited similar tumor growth inhibition, regardless of their PARP-1 trapping potency (220). Clinical studies have shown that Veliparib, which has the lowest PARP-trapping activity, can effectively treat platinum-resistant or partially platinum-sensitive BRCA-mutated epithelial ovarian cancer, with a response rate comparable to that of other PARPi (226). The future of PARP trapping as a therapeutic strategy in cancer treatment is still unfolding, and further research is needed to fully understand its potential and limitations and how it is regulated by additional proteins or cofactors.

## PARPI RESISTANCE IN THE CONTEXT OF COVALENT PARYLATION AND NON-COVALENT PAR binding

### Genome-wide and targeted screenings as a new way to identify synthetic lethal and viability targets

**Figure 4.**
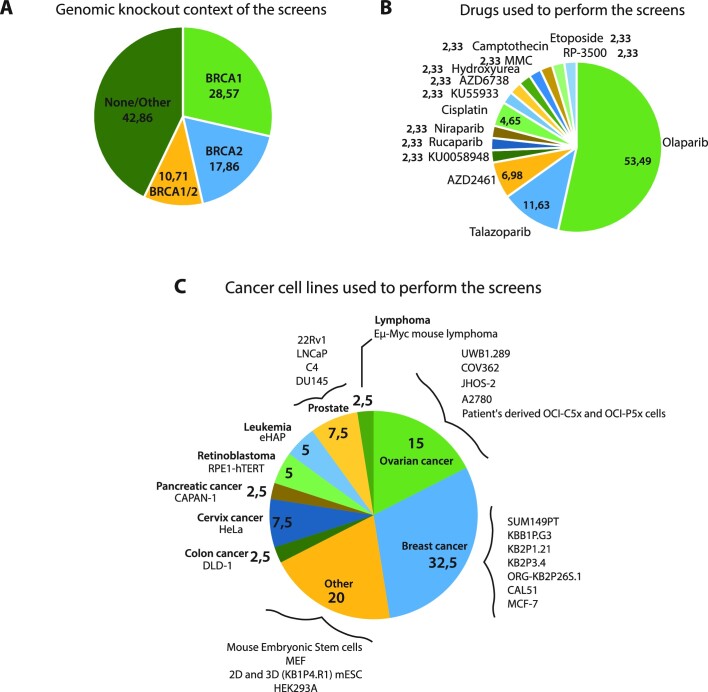
Venn diagrams of (**A**) the genomic knockout context; (**B**) drugs used; (**C**) cancer cell lines performed in whole-genome or targeted screens for genetic determinants of the PARP inhibitor response. Figure 4 is related to Table 1.

Even though the use of PARPi in the clinic has been revolutionary for thousands of patients, resistance is likely to occur in 40–70% of cancers (57) treated with these drugs and genes contributing to PARPi resistance have yet to be discovered or understood fully. To date, there are 68 clinical trials worldwide targeting PARPi-resistant cancers (ClinicalTrials.gov). RNA interference (RNAi) and CRISPR-Cas9 screen approaches have been developed to clarify synthetic lethality and viability/resistance mechanisms to PARPi with the main objective of enhancing PARPi-based therapy and overcoming therapy resistance (Figure 4 and Table 1). These screens have mainly been carried out in a BRCA1- or BRCA2-deficient context, using *in cellulo* or *in vivo* mouse models for breast or ovarian cancer. In 2018, a series of elegant screens for PARPi resistance factors have identified several resection antagonists whose loss leads to PARPi resistance in BRCA1-deficient cells, including components of the CTC1-STN1-TEN1 (CST) complex (227,228), the Shieldin (SHLD) complex (SHLD1, SHLD2, SHLD3 and REV7) (228,229), and DYNLL1 (230). These targets were also identified in a whole-genome CRISPR-Cas9 screen performed in BRCA1 mutant mouse embryonic fibroblasts (MEFs), along with Trp53bp1 (53BP1), a well-characterized protein known to antagonize HR, and the less characterized gene Thap1, the murine homolog of the transcription factor THAP1, whose loss was found to cause PARPi resistance (231). PARPi resistance upon THAP1 loss was confirmed in BRCA1 null human RPE1 cells and attributed to the rescue of HR via a mechanism that potentially involves THAP1-dependent transcription of the SHLD1 gene. A siRNA screen targeting F-box proteins by Michele Pagano's group showed that downregulation of EMI1/FBXO5 in BRCA1-deficient triple-negative breast cancer (TNBC) cells conferred PARPi resistance (232). EMI1 depletion was proposed to cause PARPi resistance by affecting ubiquitin-mediated degradation of RAD51, thereby restoring HR due to enhanced RAD51 accumulation, and by blocking mitotic entry (232,233). CRISPR-Cas9 mutagenesis screens searching for mutations in PARP-1 that would, in a BRCA1-deficient context, lead to PARPi resistance have been also described. With a tailored technique to identify in-frame mutations, Pettitt *et al.*, found mutations, both within and outside of the PARP-1 DNA-binding zinc-finger domains, that cause PARPi resistance and alter PARP-1 trapping. Of clinical relevance, they identified a mutation, R591C (WRG domain of PARP-1) in a patient with *de novo* resistance to olaparib that could abolish PARP-1 trapping and potentially contribute to PARPi resistance (234).

**Table 1.** Whole-genome or targeted screens for genetic determinants for PARP inhibitor response

			Screen context				Drug response			Cell line used and genetic status			PAR status	
Reference	DOI	Year of publication	BRCA1-/-	BRCA2-/-	Targets	Library	PARPi	Other drug	PARPi response upon gene loss	Cell line	Genetic status	Cell type	Non covalent PAR binding	Covalent PARylation
He *et al.*	doi:10.1038/s41586-018-0670-5	2018	**✓**	X	DYNLL1	Updated GeCKO library	Olaparib	Cisplatin	Resistant	UWB1.289	BRCA1-mut	Ovarian cancer		**✓**
										COV362	BRCA1-mut	Ovarian cancer		
										JHOS-2	BRCA1-mut	Ovarian cancer		
Pettitt *et al.*	doi: 10.1038/s41467-018-03917-2	2018	**✓**	X	PARP1mut	Tzelepis *et al.*, 2016	Talazoparib	**✓**	Resistant	SUM149PT	BRCA1-mut	Breast cancer	**✓**	**✓**
										Mouse ES cells	-	Embryonic Stem cells		
Barazas *et al.*	https://doi.org/10.1016/j.celrep.2018.04.046	2018	✓	X	CTC1	Costantino *et al.*, 2014; Xu *et al.*, 2015	Olaparib/ Talazoparib/ AZD2461	✓	Resistant	KBB1P.G3	BRCA1-mut	Breast cancer		
										Mouse ES cells	BRCA1-mut	Embryonic Stem cells		
										SUM149PT	BRCA1-mut	Breast cancer		
Noordermeer *et al.*	doi:10.1038/s41586-018-0340-7	2018	✓	X	SHLD complex	TKOv1 sgRNA library	Olaparib/ Talazoparib	✓	Resistant	RPE1-hTERT TP53-/-	BRCA1-/-	Retinoblastoma		
										SUM149PT	BRCA1-mut	Breast cancer		
Dev *et al.*	doi:10.1038/s41556-018-0140-1	2018	✓	X	c20orf196 (SHLD1)	GeCKOv2	Olaparib/ Talazoparib/ ADZ2461	✓	Resistant	SUM149PT	BRCA1-mut	Breast cancer		
					FAM35A (SHLD2)				Resistant					
Marzio *et al.*	doi:10.1016/j.molcel.2018.11.003	2019	✓	X	EMI1	69 siRNA SMARTpool library againt human F-BOX69 siRNA SMARTpool library againt human F-BOX69 siRNA SMARTpool library againt human F-BOX	Olaparib	✓	Resistant	SUM149PT	BRCA1-mut	Breast cancer		✓
Shinoda *et al.*	doi:10.1016/j.molcel.2021.03.034	2021	X	✓	THAP	Genome-scale mouse Brie CRISPR knockout pooled library	Olaparib	✓	Resistant	MEF	BRCA1-mut +/-p53	Mouse embryonic fibroblasts		
Paes Dias *et al.*	https://doi.org/10.1016/j.molcel.2021.09.005	2021	X	✓	LIG3	pLKO.1; DDR library derived from the Sigma Mission library (TRCMm1.0) (lentiviral hairpins)	Olaparib	✓	Sensitive	2D and 3D (KB1P4.R1) mESC	TP53-/- TP53BP1-/-	Mice Embryonic Stem Cells	✓	✓
Gogola *et al.*	doi:10.1016/j.ccell.2018.05.008	2018	X	✓	PARG	1976 shRNA custom library	Olaparib/ AZD2461	✓	Resistant	KB2P1.21	BRCA2-/-	Breast cancer	✓	✓
										KB2P3.4	BRCA2-/-	Breast cancer		
										ORG-KB2P26S.1	BRCA2-/-	Breast cancer		
Mengwasser *et al.*	doi:10.1016/j.molcel.2018.12.008.	2019	X	✓	FEN1	Custom shRNA library	✓	✓	Sensitive	DLD-1	BRCA2-mut	Colon adenocarcinoma	✓	✓
					APEX2				Sensitive					✓
Clements *et al.*	doi:10.1038/s41467-020-19961-w	2020	X	✓	HUWE1	Brunello Human CRISPR knockout pooled library	Olaparib	✓	Resistant	HeLa	BRCA2-/-	Cervix adenocarcinoma	✓	✓
					KAT5				Resistant					
Kharat *et al.*	doi:10.1126/scisignal.aba8091	2020	✓	✓	TET2	Ambion Silencer Mouse Genome siRNA collection	Olaparib	-	Resistant	mESC	Hypomorphic BRCA2	Mice Embryonic Stem Cells		
Tang *et al.*	doi:10.1093/nar/gkab540	2021	✓	✓	Cyclin C	TKOv3 gRNA library	Olaparib	✓	Resistant	HEK293A	BRCA2-/-	Human Embryonic Kidney cells		
Turner *et al.*	doi:10.1038/emboj.2008.61	2008	✓	✓	CDK5	siARRAY, targeting 779 known and putative human protein kinase genes	KU0058948	✓	Sensitive	CAL51	BRCA2-mut +/- siBRCA1	Breast cancer		✓
Bajrami *et al.*	doi:10.1158/0008-5472.CAN-13-2541	2014	✓	✓	CDK12	OpenBiosystems GIPZ human shRNA library	Olaparib	✓	Sensitive	MCF-7	-	Breast cancer		✓
Verma *et al.*	doi:10.1038/s41556-020-00624-3.	2021	✓	✓	ALC1	Domain-focused sgRNA pooled library (custom)	Olaparib/ Talazoparib	✓	Sensitive	CAPAN-1	BRCA2-mut	Pancreatic adenocarcinoma	✓	✓
										SUM149PT	BRCA1-mut	Breast cancer		
										UWB1.289	BRCA1-mut	Ovarian cancer		
Zimmermann *et al.*	doi:10.1038/s41586-018-0291-z	2018	-	-	RNAseH2	TKOv1 virus library	Olaparib	✓	Sensitive	HeLa	-	Cervix adenocarcinoma	✓	✓
										RPE1-hTERT	-	Retinoblastoma		
										SUM149PT	BRCA1-mut	Breast cancer		
Fang *et al.*	doi:10.1038/s42003-019-0580-6	2019	-	-	TIGAR	GeCKO v2.0 pooled libraries	Olaparib	✓	Sensitive	A2780	-	Ovarian cancer		
Lui *et al.*	doi:10.1016/j.ebiom.2020.102988	2020	-	-	BRD4	Custom siRNA library	Rucaparib	✓	Sensitive	Patient's derived OCI-C5x and OCI-P5x cells	Some BRCA1-mut	Ovarian cancer		✓
Juhasz *et al.*	doi:10.1126/sciadv.abb8626	2020	-	-	ALC1	GeCKOv2	Olaparib	✓	Sensitive	HeLa	-	Cervix adenocarcinoma	✓	✓
Su *et al.*	doi:10.1016/j.dnarep.2020.102803	2020	-	-	POLE3/4	DDR-focused CRISPR library targeting 365 genes (custom)	Olaparib	KU55933, AZD6738, LY2606368, hydroxyurea, cisplatin, MMC, camptothecin and etoposide	Sensitive	HEK293A	TP53-/-	Human Embryonic Kidney cells		
Hewitt *et al.*	doi:10.1016/j.molcel.2020.12.006	2021	-	-	BRCA2	Brunello Human CRISPR knockout pooled library	Olaparib	✓	Sensitive	eHAP	ALC1-/-	Chronic myelogenous leukemia cells		✓
					ATM				Sensitive				✓	✓
					UBC13				Sensitive				✓	✓
					SMUG1				Resistant					
Fugger *et al.*	doi:10.1126/science.abb4542	2021	-	-	DNPH1	Brunello Human CRISPR knockout pooled library	Olaparib	✓	Sensitive	eHAP	MUS81 -/-	Chronic myelogenous leukemia cells		
Ipsen *et al.*	doi:10.1038/s41388-022-02427-2	2022	-	-	PARP-1	Brunello Human CRISPR knockout pooled library	Olaparib	✓	Resistant	C4	-	Castration-resistant prostate cancer	✓	✓
					ARH3				Resistant				✓	
					YWHAE				Resistant				✓	✓
					UBR5				Resistant					
Zhang *et al.*	doi:10.3389/fonc.2022.999302	2022	-	-	TBL1XR1	Cellecta KOHGW-80K-P library	Olaparib	✓	Sensitive	22Rv1	-	Prostate carcinoma	✓	
Xu *et al.*	doi: 10.1158/1541-7786.MCR-20-0791	2022	-	-	CHK2	Custom pooled DNA repair lentiviral sgRNA library	Olaparib	-	Resistant	Eμ-Myc mouse lymphoma	-	B-cell lymphoma		
Zimmermann *et al.*	https://doi.org/10.1016/j.celrep.2022.111081	2022	-	-	RNASEH2B	TKOv3 sgRNA library	Niraparib	RP-3500 (ATRi)	Sensitive	RPE1-hTERT TP53-/-	TP53-/-	Human Embryonic Kidney Cells		✓
					RAD51B				Sensitive					
					RAD51C				Sensitive					
					RAD51D				Sensitive					
Tsujino *et al.*	doi:10.1038/s41467-023-35880-y	2023	-	-	MMS22L	CRISPR-Cas9 KO H1 and H2 libraries obtained from Drs. Myles Brown and X. Shirley Liu's laboratories	Olaparib	✓	Resistant	LNCaP, C4-2B, 22Rv1 and DU145 cells	DU145: BRCA2d12	Prostate carcinoma		
					CHK2				Resistant					
					E2F7				Resistant					✓

In a BRCA2-deficient context, screening of mouse mammary tumor-derived cultures, 2D cell lines and 3D organoids by Gogola *et al.* identified loss of PARG as a contributor to PARPi resistance (235). The role of PARG loss in PARPi resistance will be further detailed below because it is now recognized as a potential PARPi resistance mechanism in human cancers. Using DNA repair-focused shRNA and CRISPR-based libraries, Mengwasser *et al.* identified endonucleases FEN1 and APEX2 as BRCA2 synthetic lethal targets and these candidates were also found to be synthetic lethal with BRCA1 during the downstream validation process (236). Genome-wide CRISPR screens by Clements *et al.* reported that the ubiquitin ligase HUWE1 and the histone acetyltransferase KAT5/TIP60 cause resistance to PARPi when depleted in BRCA2-deficient cells. Loss of HUWE1 was proposed to cause PARPi resistance by partially restoring HR via an increase in RAD51 levels while KAT5 depletion was shown to promote 53BP1 binding to DSBs, leading to a reduction in DNA end resection and subsequent PARPi resistance, potentially through promoting DSB repair by NHEJ (237). Another whole-genome CRISPR screen identified cyclin C (CCNC) and RNA Pol II transcription mediator complex components as synthetic survival targets, i.e. their loss led to improved survival and PARPi resistance in BRCA2-depleted cells, most likely via a mechanism of stabilization of replication forks (238). A genome-wide siRNA screen carried out in PARPi-sensitive mESCs expressing a hypomorphic allele of BRCA2 identified DNA demethylase TET2 as a gene whose loss conferred olaparib resistance and established a link between epigenetic regulation of DNA and PARPi resistance (239).

Several screens have also been performed in a non-BRCA context. Many of these have identified genes whose inactivation causes PARPi sensitization, making them potential combinatorial targets with PARPi. Zimmermann *et al.* performed CRISPR-KO screens in three different cell lines (HeLa, RPE-hTERT and SUM149PT) and discovered 73 genes that cause increased sensitivity to PARPi when mutated, including ribonuclease H2 (RNASEH2) whose loss sensitized cells to PARPi regardless of the BRCA status. Further investigations in RNaseH2-deficient cells suggested that the underlying cause of the PARPi hypersensitivity was a result of impaired ribonucleotide excision repair (RER), resulting in PARP-trapping lesions that block DNA replication and compromise genome integrity (240). TIGAR is another PARPi response modifier that has emerged from a CRISPR-Cas9 screen using PARPi. Its knockdown has been proposed to induce ‘BRCAness’ (241) by downregulation of BRCA1 and the Fanconi anemia pathway, thereby sensitizing cancer cells to olaparib (242). A genome-wide CRISPR knockout screen in HeLa cells by Juhasz *et al.* showed that loss of the PAR–dependent chromatin remodeler ALC1 increased sensitivity to PARPi. ALC1 deficiency enhanced PARP-1 trapping, then impairing the binding of NHEJ and HR repair factors to DNA lesions and subsequently causing PARPi sensitivity (243). ALC1 also emerged as a gene whose loss conferred PARPi sensitivity in a domain-focused CRISPR screen involving BRCA1- and BRCA2-mutant cells (244). Interestingly, a genome-wide CRISPR screen in ALC1-deficient cells found that deficiencies in BRCA2, but also in several other DSB repair factors such as ATM, DNA2, UBC13/UBE2N and to a lesser extent RAD51 and RAD51C, conferred synthetic lethality and PARPi hyper-sensitization when combined with the loss of ALC1 (245). Lui *et al.* performed high-throughput RNAi screening in different patient-derived ovarian cancer cells and found that knockdown of Bromodomain-containing protein 4 (BRD4) and other components of the transcriptional machinery sensitized cells to rucaparib (246). Su *et al.* conducted DDR-focused CRISPR screens in HEK293A cells and observed that loss of the two subunits of DNA polymerase epsilon, POLE3/4, sensitized cells not only to olaparib, but also to an ATR inhibitor and camptothecin (247). Fugger *et al.* also performed a genome-wide CRISPR screen in HR-deficient MUS81^−/−^ cells and identified DNPH1 (2'-deoxynucleoside 5'-phosphate N-hydrolase 1), a protein that eliminates cytotoxic nucleotide 5'-hydroxymethyl-deoxyuridine (hmdU) monophosphate, as top hit that caused hypersensitivity to PARPi. Inhibition of DNPH1 resensitized PARPi-resistant BRCA-deficient cells to PARPi (248).

Over the last few years, several genome-wide CRISPR screens to reveal the genetic determinants of PARPi response in prostate cancer have been performed. Ipsen *et al.*, identified and validated three DNA repair-associated genes, ARH3, YWHAE and UBR5, along with PARP-1 as novel candidates associated with PARPi resistance upon knockout in the C4 castration-resistant prostate cancer (CRPC) cell line (249). Zhang *et al.* screened 3D spheroids from an olaparib-insensitive cell line and demonstrated that deficiency in TBL1XR1, a core component of nuclear receptor corepressor, sensitized prostate cancer cells to PARPi (250). Screens by Tsujino *et al.* in BRCA1/2-proficient prostate cancer cells identifed the DNA repair gene MMS22L, whose loss hypersensitized cells to PARPi, presumably by disrupting RAD51 recruitment to PARPi-induced DSBs and causing HR deficiency (251).

Unsurprisingly, a significant proportion of these targets sharing a synthetic lethal relationship with PARPi, are PAR readers themselves [SHLD2 (OB-fold); 53BP1 (BRCT and GAR); HUWE1 (WWE domain); ALC1 (Macrodomain)], partners of PAR readers [DYNLL1 binds MRE11 (43); UBE2N binds RNF8 and RNF168 (RING and FHA domains); FBXO5 and TBL1XR1 relates to the F-box/WD40 protein family (191); FEN1 binds BLM and WRN (252); UBR5 binds TOPBP1 (BRCT)]; or involved in the turnover of the PARylation process [PARG and ARH3] (6).

In summary, genome-wide CRISPR-Cas9 and RNAi screens are powerful tools for identifying biomarkers and mechanisms of sensitivity and resistance to PARPi. The resulting data are paving the way to a better understanding and prediction of patient responses to PARPi as well as to the design of new inhibitors that may help overcome PARPi resistance. Targeting PAR–protein interactions by PAR readers in the DDR network is a promising but underutilized strategy for improving PARPi efficacy. Disrupting PAR–protein interactions might be an avenue for drug design. However, developing effective and specific small-molecule PAR–protein interaction inhibitors might be challenging. Still, targeting protein interactions in the DDR pathway has provided multiple opportunities for the development of cancer therapies (253). In fact, there are multiple examples of DDR proteins having functionally druggable PAR recognition modules. One has just to think about the variety of DDR factors relocalized to DNA lesions in a PAR-dependent fashion. A disruption of the PAR–protein interface would likely prevent the proper functioning of the target in cells. For example, ALC1 relies on its PAR binding activity to remodel chromatin during the DDR. A disruption of this essential component is likely to enhance the sensitivity of HR-deficient cells to PARPi, similar to what has been shown for ALC1 depletion (244,254).

### Resistance mechanism to PARPi

PARPi resistance mechanisms have been divided into four main categories: (i) HR restoration, (ii) changes in PARP-1 activity and PAR levels, (iii) cellular availability of PARPi and (iv) restoration of replication fork protection. Nonetheless, other mechanisms are rapidly emerging (255). Here, we review the best-described PARPi resistance mechanisms with a focus on HR deficiency caused by BRCA1/2 alterations and include the newly discovered mechanism of ssDNA gap suppression.

#### Restoration of HR

HR deficiency is prevalent in a wide range of cancers, presumably afflicting approximately 50% of high-grade epithelial ovarian cancers (EOC), and is being actively investigated as actionable vulnerability, namely for its potential for sensitizing cancer cells to platinum-based DNA-damaging chemotherapy (e.g. cisplatin and carboplatin) and at promoting synthetic lethality with PARPi treatment (256–258). The ability of PARPi to selectively eradicate HR-deficient cancer cells was first illustrated in cells lacking HR components BRCA1 and BRCA2 and provided the basis for the clinical development of PARPi (259,260). Since a growing number of genetic or epigenetic alterations in other HR-related genes, including ATM, ATR, PALB2, BARD1, BRIP1, RAD51B, RAD51C, RAD51D, FANCA, and non *bona fide* HR genes, such as PTEN and CDK12, have been linked to HR deficiency with preclinical or clinical evidence of sensitivity to PARPi and/or DNA-damaging agents, extending the clinical use of these treatments beyond BRCA1/2 defects (261). Classically, the antitumor activity of PARPi in an HR-defective background has been primarily attributed to their ability to trap PARP-1 on damaged DNA, resulting in replication fork collapse and subsequent generation of DSBs that can only be repaired by alternative, error-prone pathways including NHEJ and single-strand annealing (SSA) (262,263). Repair of resulting DSBs by mutagenic pathways instead of error-free HR causes genomic instability leading to cell death. In this line, restoration of HR is one mechanism leading to PARPi resistance with the most clinical evidence and it can occur via several routes. Secondary reversion mutations in key HR genes, BRCA1, BRCA2, PALB2, RAD51C or RAD51D, restoring the original open reading frame (ORF), consequently restoring protein expression and function, have been described in patients (264–268). Mechanisms that restore BRCA, other than secondary reversion mutations (e.g. alternative splicing, alternative translation initiation, copy number gain and/or upregulation of the remaining functional allele), that stabilize BRCA1-mutant proteins, and that upregulate HR and HR-associated genes are other potential resistance mechanisms (269–273). For instance, the upregulation of RAD51, the central recombination enzyme, is a common feature in BRCA1-deficient tumors and is associated with poor patient outcome and PARPi resistance (274,275). One mechanism by which RAD51 can become upregulated in tumors is through the downregulation of EMI1/FBXO5. Work by Marzio *et al.* showed that EMI1/FBXO5 constitutively controls ubiquitin-mediated degradation of RAD51 and suggested that a subset of BRCA1-deficient triple-negative breast cancer (TNBC) cells develop resistance to PARPi due to reduced levels of EMI1/FBXO5, causing accumulation of RAD51 and consequently restoring HR (232). When HR is functional, RAD51 assembles at DNA damage sites into nuclear foci that can be visualized by indirect immunofluorescence in cells or by immunostaining on PDX samples (276,277). Detection of RAD51 foci has emerged as a promising biomarker of HR proficiency and PARPi resistance in different types of cancers regardless of the underlying HR restoration mechanism (277).

Epigenetic modifications of HR-associated genes have also been shown to influence PARPi resistance. Namely, the silencing of BRCA1 and RAD51C by promoter methylation has been associated with HR deficiency and PARPi sensitivity in both clinical and preclinical models (256,267,278,279). Accordingly, BRCA1 and RAD51C methylation loss has been linked to resistance in HGSC patients and patient-derived xenograft (PDX) models, respectively (280,281).

Another mechanism of HR restoration includes suppression of NHEJ in BRCA1-deficient cells. In normal cells, BRCA1 and 53BP1 act antagonistically to maintain a balance between HR and NHEJ and this balance is shifted toward error-prone NHEJ in BRCA1-deficient cells (282). 53BP1 promotes NHEJ by preventing extensive DNA end resection, a crucial step for HR repair and it does so by interacting with RIF1 and the Shieldin complex (SHLD1, SHLD2, Rev7, SHLD3) (228,283–285). Loss of 53BP1 and components of the 53BP1-RIF1-Shieldin complex has been shown to reactivate DNA end resection and rescue HR in BRCA1-deficient cells (286). The 53BP1-RIF1-Shieldin complex was found to counteract DSB resection through CST/ Polα-dependent fill-in of DSB ends. Consistent with this, CST depletion leads to increased resection in a manner similar to the loss of 53BP1/Rif1/Shieldin (287). DYNLL1 is another example of a resection inhibitor whose loss causes PARPi resistance in BRCA1-deficient (230).

#### Changes in PARP-1 activity and PAR levels

Loss of PARP-1 function has been linked to the development of PARPi resistance both preclinically and clinically. As mentioned above, PARP-1 mutation R591C, recently identified in the tumour of a PARPi-resistant patient, was found to prevent PARP-1 trapping, providing the first clinical evidence linking PARPi resistance with loss of PARP-trapping ability (234). PARG is the main PAR degrading enzyme and its loss has been shown to restore PAR formation in PARPi-treated cells and partially rescue PARP-1 signalling, resulting in PARPi resistance (235,288). Phosphorylation of PARP-1 at Y907 by the tyrosine kinase c-Met has been reported to increase PARP-1 catalytic activity and reduce the binding of PARPi, thereby causing PARPi resistance in cancer cells (289). Recently, a heterodimer of EGFR and MET was found to interact with and phosphorylate Y907 contributing to PARPi resistance in TNBC cells (290). Overexpression of PARP-1-binding partners, p97/VCP and HMGB3, which have been shown to remove cytotoxic trapped PARP-1–DNA complexes, and overexpression of ALC1, which can remove inactive PARP-1 indirectly through binding to PARylated chromatin, are mechanisms found in different types of tumors and that have been linked to PARPi resistance (224,291).

In general, any factor stimulating the activity of DNA-dependent PARPs, even in a DNA-independent way, is likely to promote PAR accumulation in cells. For example, TSG101 (Tumor susceptibility gene 101 protein) is essential for cellular PARylation (292). Depletion of TSG101 causes PARP-1 trapping at DNA damage foci, an observation that suggests that stimulatory factors of PARP-1 activity might be promising targets to improve the potency of PARPi. Other factors, such as the C_2_H_2_-type zinc finger protein CTCF stimulates PARP-1 (293), which increases cellular PAR levels but also becomes covalently PARylated. PARylation of CTCF is essential for recruiting BRCA2 to DSBs (187,294). As aforementioned, the levels of PARylation correlate with PARPi sensitivity in ovarian cancers (225). The global dynamics of PARylation between PAR writers, assisted by their stimulatory factors, and PAR erasers, operating under a specific genetic background, contributes to defining the cellular levels of PAR and the clinical outcomes. Finally, it should be emphasized that not only the levels of PARylation could determine the sensitivity to PARPi but also the amino acid-specific PARylation patterns (158). Although HPF1-dependent serine-PARylation is essential to the DDR (145,295), the contribution of ASP/GLU-PARylation could be underappreciated, and the interplay between both types of ADP-ribose conjugation systems remains unclear. A PARylation code could presumably define PAR turnover, especially in a system that involves amino acid-specific ADP-ribose erasers (6,62,64).

#### Cellular availability of PARPi

ATP-binding cassette (ABC) transporters mediate the efflux of multiple chemotherapeutic drugs and are well-known causes of multidrug resistance (MDR) in human cancers when overexpressed. Accumulating evidence suggests that PARPi resistance may be caused by a reduction of intracellular PARPi concentration via overexpression of the drug efflux transporter P-glycoprotein (P-gp), also known as MDR1 and encoded by the ABC transporter subfamily B member 1 (ABCB1) gene. All four FDA-approved PARPi are substrates of the P-gp efflux transporter and overexpression of the latter has been linked to olaparib resistance in cell lines, animal models and samples from patients with resistant high-grade serous ovarian cancer (HGSC) (296–300). Several P-gp inhibitors have been shown to reduce resistance to olaparib in preclinical studies, but investigation in clinical trials has yielded poor outcomes (272,300). Efforts to develop optimized MDR reversing agents, such as olaparib conjugates and PARPi that are not substrates of ABC transporters are ongoing (301,302).

#### Restoration of replication fork protection

Trapped PARP-1-DNA complexes that result from PARPi treatment pose as obstacles to replication. When a replication fork encounters an obstacle in normal cells, it may stall and undergo reversal as a protective mechanism to allow time for repair and restart. Replication fork reversal involves remodeling of the stalled replication fork into a four-way junction that requires protection from degradation by nucleases such as MRE11 and MUS81 (303). Apart from their role in HR, BRCA1/2, RAD51, and components of the FA pathway as FANCD2 are important players in the protection of reversed replication forks (304). In the absence of BRCA1/2, fork protection is alleviated. MRE11 is then recruited to forks in a manner that depends namely on PARP-1, PTIP, and CHD4, while MUS81 recruitment occurs in a EZH2-dependent manner, leading to extensive fork degradation and subsequent collapse into deadly DSBs (305). Therefore, events that restore replication fork protection are likely to cause PARPi resistance. In agreement with this, disruption of PARP-1, PTIP, CHD4, MUS81, or EZH2 restored fork protection by preventing nuclease degradation, conferring PARPi resistance in BRCA-defective cells (306,307). Similarly, a microRNA, miR-493–5p, downregulates MRE11, CHD4 and EXO1 in BRCA2 mutant cells and was found to protect the replication fork from nuclease degradation and induce PARPi resistance (308). Inactivation of SNF2-family fork remodelers SMARCAL1, ZRANB3, and HLTF also protected stalled forks from MRE11-dependent degradation in BRCA1/2-deficient, causing resistance to PARPi (309). FANCD2 overexpression, which has been reported in different types of cancers, was found to confer resistance to PARPi by stabilizing replication forks in BRCA1/2-mutant cells (310). RADX is an RPA-like, single-strand DNA binding protein recruited to replication forks, where it antagonizes the accumulation of RAD51 to inhibit inappropriate fork reversal. RADX deletion restored fork protection in cancer cells lacking BRCA2 (311,312). SLFN11, which has been shown to induce a lethal replication block in response to PARPi, is another factor whose loss conferred PARPi resistance in BRCA1/2-deficient cells (313,314). Work by Kharat *et al.* has linked DNA demethylase TET2, which catalyzes the conversion of DNA methylation mark 5-methylcytosine (5mC) to 5-hydroxymethycytosine (5hmC), to the degradation of stalled replication forks. The epigenetic mark 5hmC left by TET2, when at stalled replication forks, recruits the endonuclease APE1. Loss of TET2, which has been observed in several malignancies, has been shown to promote PARPi resistance by protecting replication forks, presumably as a result of a decreased degradation of 5hmC-marked stalled replication forks by APE1 in BRCA2-deficient cells (239).

#### Suppression of ssDNA GAPS

For many years, the synthetic lethality between PARPi and BRCA1/2 has been attributed to DSBs arising from defects in HR and/or fork protection, but accumulating evidence is proposing ssDNA gaps as the primary toxic lesion promoting PARPi sensitivity in BRCA-deficient cells. According to this recent model, PARPi resistance can emerge from reducing DNA replication gaps via regain of Okazaki fragment processing (OFP) (315). This and other mechanisms of ssDNA gap suppression have been reviewed in more detail by Jackson and Moldovan (316). Although preclinical investigations have revealed an increasing number of plausible PARPi resistance mechanisms, it is important to note that, for many, clinical relevance has yet to be determined. Because *in cellulo* findings may not always translate clinically, further analysis of resistance mechanisms through patient studies is necessary for a proper understanding of the determinants of PARPi response. Clinical studies with larger cohorts and improved methods for monitoring resistance in tumour material will be needed to confirm and clarify the resistance mechanisms highlighted by preclinical studies. In particular, analysis of cell-free DNA (cfDNA) in liquid biopsy is a non-invasive, low-cost, and promising alternative to tumor biopsy that is emerging to capture resistance patterns and may enhance precision care (317).

### Overcoming resistance of cancer cells to PARPi using drug combinations

The occurrence of drug resistance has emphasized the need for combination approaches that would work synergistically to enhance anti-tumour activity over single agents and allow the use of lower doses to minimize toxicity. Over the recent years, strategies are gradually evolving to combine PARPi with DDR inhibitors targeting two main classes of molecules, that is cell-cycle checkpoint and DSB repair factors.

Inhibitors of cell-cycle checkpoint kinases ATR, CHK1 and WEE1 have differential capacities to alter the restoration of HR and/or protection of stalled replication forks, two mechanisms associated with drug resistance in HR deficient tumors. The ATR–CHK1–WEE1 pathway has produced several clinical inhibitor candidates that are currently undergoing clinical development as a single agent or in combination with PARPi, including novel ATRi RP-3500, a highly potent, selective, and orally bioavailable inhibitor that has shown preclinical promise in BRCA1/2- or ATM-deficiency (318,319). Recently, Zimmerman and colleagues performed a genome-wide chemogenomic CRISPR screen in RPE1-hTERT TP53 knockout cells to identify genetic alterations that sensitized cells to a combination of PARPi with ATRi RP-3500. They found alterations in ATM, RNASEH2A, RNASEH2B, RNASEH2C, and RAD51 paralogs RAD51B and RAD51D to be particularly sensitive to the PARPi-ATRi combination, providing precious insights to guide the choice of combination strategies (320).

Within the DNA repair network, several druggable targets, such as RAD51, POLθ, RAD52, PARG and CDK12, have potent inhibitors in preclinical or clinical development and may serve as potential synthetic lethal partners for PARPi in targeting HR alterations (321,322). Of interest, RAD52 and POLθ have been proposed to mediate parallel backup DSB repair pathways, i.e. RAD52-dependent HR and single-strand annealing (SSA) in the case of RAD52 and theta-mediated end-joining (TMEJ) or A-NHEJ for POLθ, that provide potential escape routes from the toxic accumulation of endogenous and drug-induced DNA damage in HR-deficient cells. Thus there has been considerable optimism about using RAD52 and POLθ inhibitors alone, or in combination strategies to block these routes and potentiate PARPi and other DNA-damaging treatments in HR-deficient backgrounds (263,323). A small molecule targeting POLθ has been shown to be synthetic lethal with BRCA1- and BRCA2-deficient cells, but also to reverse PARPi resistance caused by 53BP1/SHLD defect (324). Another POLθ inhibitor, ART4215, is being clinically evaluated in combination with talazoparib in participants with advanced or metastatic solid tumors. Although not a DNA repair factor *per se*, CDK12 is a transcriptional regulator of DDR whose inactivation promotes BRCAness, thus conferring sensitivity to PARPi (325). Several other strategies that exploit pharmacologic induction of BRCAness with targeted agents, such as PI3K/AKT/mTOR pathway inhibitors, tyrosine kinase inhibitors, CDK inhibitors, BET inhibitors, or DDR inhibitors, are being studied in combination with PARPi to enhance cancer therapy and are more extensively reviewed elsewhere (326).

As there is increasing evidence linking PARPi resistance with the restoration of PARylation or loss of PARP-trapping ability, strategies that alter PAR signaling are also being evaluated in combination with PARPi. Namely, PARPi resistance mediated by the c-MET/pY907 PARP-1 axis, known to increase PARP-1 catalytic activity and reduce PARPi binding, has been demonstrated in TNBC and HGSOC cells, and the combination of PARPi fluzoparib (HS10160) and METi (HS10241) has shown synergism in these cell lines. These results have suggested that the level of Y907 phosphorylation of PARP-1 may serve as a biomarker to predict PARPi resistance and that the combination of c-Met and PARPi may benefit patients with high c-Met expression tumours (290,327). While METi (HS10241) is under clinical investigation as single agents in solid tumors, the combination of PARPi and METi, although promising, has not been examined clinically yet (327). Blocking c-Met and EGRF, known to interact with the c-MET/pY907 PARP-1 axis, was shown to reverse PARPi resistance in TNBC cells, suggesting that combined inhibition of c-MET and EGFR could also be exploited to sensitize TNBC to PARPi (290). Recently, inhibition of two PARP-1-binding partners, p97/VCP and HMGB3, has been shown to cause PARPi sensitization by prolonging PARP trapping with DNA, delineating new combination approaches to enhance PARPi cytotoxicity. Small-molecule inhibitors that target p97, such as a metabolite of the clinically used disulfiram (CuET) and the orally bioavailable CB-5083, considerably enhanced PARPi sensitivity in HR-defective tumour cells and patient-derived tumour organoids, and may have clinical potential. Work by Stephen West *et al.* has demonstrated that targeting the nucleotide salvage factor DNPH1, which eliminates cytotoxic nucleotide 5'-hydroxymethyl-deoxyuridine (hmdU) monophosphate, can hypersensitize HR-deficient cells to PARPi (248). The group reported that DNPH1 inhibition increased hmdU, promoting PARP trapping, DSB formation, and cell death through the SMUG1 glycosylase. This finding might drive the development of DNPH1 inhibitors.

The recently discovered role of PARPi in regulating immune responses has prompted the evaluation of several approaches combining immune checkpoint inhibitors (ICIs), such as PD-1 or PD-L1 inhibitors, and PARPi. The combination of PARPi with ICIs has demonstrated a significant synergism in preclinical models and is currently being investigated clinically (328–330).

DDR-associated drug discovery has mainly focused on catalytic inhibitors that block enzyme active sites, which limits the number of potential drug targets within the DDR pathways (253). In that perspective, PAR binding proteins and PARylated substrates deepen the pool of druggable targets. Most studies relied on genetic screening to identify biomarkers of resistance or sensitivity to DNA repair inhibitors. Rather than a gene-centered view of PARPi sensitivity and resistance, a growing body of work suggests that PAR readers and covalently PARylated proteins work in concert with other DNA repair factors to coordinate the cellular response to DNA damage. The development of small molecule inhibitors to directly disrupt the PAR-protein interaction is a potential strategy to induce BRCAness and potentiate the cytotoxic effect of PARPi in drug combination therapies. Following this idea, MRE11 could provide an example of a clinically relevant PAR reader that could be targeted to improve the therapeutic response. MRE11 is central to the formation of the MRN complex (MRE11, NBS1, RAD50) which is essential for sensing and signaling DSBs (331). Similar to several DSB-interacting proteins, MRE11 binds PAR *in vitro* and is recruited to DNA lesions in a PAR-dependent manner (43). MRE11 was also identified using recent MS-based strategies developed to specifically identify PAR readers from covalently PARylated proteins as a non-covalent PAR binding protein in cells (33,35). The inhibition of MRE11 with Mirin (332) has been shown to sensitize cancer cells towards genotoxic agents (331) and lead to increased sensitivity to PARPi (333). A small-molecule modulator of MRE11–PAR interactions could block the recruitment of the MRN complex and downstream repair proteins and mimic MRE11 inhibition similar to Mirin. In theory, this approach could provide new opportunities to sensitize cancer cells to PARPi. Interfering with PAR binding can propagate changes through a broad web of PAR interactions. PAR acts as a hub in the DDR so a disruption of PAR-protein interactions with small molecules could change the dynamics of DDR-associated protein networks and ultimately determine cell fate. Many PARylated proteins are also likely to modulate PARP trapping ability. Several trapped PARP-1-associated proteins are both non-covalent PAR binding proteins and covalently PARylated substrates (224). At this stage, it is not clear whether a PARylated substrate or a PAR reader can directly weaken or strengthen the interaction of PARP-1 with DNA. However, one can imagine situations where the depletion or the overexpression of a PAR binding protein or a PARylated substrate could modulate the trapping of PARP-1 in DNA lesions. We hypothesize that DDR inhibitor-based combination therapies with small-molecule modulators of protein–PAR interactions could increase the cytotoxicity of PARPi.

## CONCLUSIONS AND PERSPECTIVES

PARylation is a widespread nuclear PTM that is of pivotal importance for maintaining genome integrity. The biosynthesis of diverse PAR polymers structures is based on the tight regulation and dynamic action of PAR writers and erasers, as well as a complex set of PAR readers that fine-tune DDR and DNA repair signaling. Many covalently PARylated proteins and PAR binding proteins can serve as sensitive switches to dictate the repair pathway choice. Defective DNA repair is highly prevalent in cancers and confers hypersensitivity to PARPi. Trapping PARP-1 on specific DNA lesions, including repair intermediates, is the prevailing model explaining how PARPi effectively kills HR-defective cells. Although this model is well supported by evidence, trapping is not the sole contributor to the different biological activities observed for different PARPi.

PARylation changes usually represent different meanings in terms of prognosis and potential treatment. The incomplete mechanistic understanding of PARPi-mediated toxicity currently limits the impact of PARPi-induced synthetic lethality. The identification and functional characterization of factors involved in the dynamics of PARylation will provide new perspectives and opportunities to increase PARPi sensitivity and may identify potential strategies with drugs that target the DDR. This concept has been overlooked and underresearched because, until recently, it was difficult to uncover its precise nature on a proteome-wide scale and to separate non-covalent PAR binding from covalent PARylation. Thanks to recent methodological developments in MS-based ADP-ribose proteomics analysis, the distinction between the two modes of PAR association can be made although a high degree of functional overlap is common. The dysregulation of the PARylation dynamics through the development of small molecules modulators of PAR–protein interactions could be an attractive strategy to affect the progression of a variety of cancers since PAR is a master orchestrator of the DDR pathway which confers cancer-specific vulnerabilities. The complexity of a highly dynamic system such as PARylation present many challenges for the development of druggable molecules with both selectivity and potency. PAR readers associate with PAR through unique recognition patterns and small peptides can exhibit high PAR binding affinity. These two characteristics should facilitate the development of specific PAR–protein modulators and broaden the therapeutic landscape of PARPi.

PARylation is at the heart of clinical activity in patients harboring HR-deficient tumors. Although PARylation contains MS-labile bonds, presents molecular heterogeneity and is often of low abundance, it is at the center of a diverse array of biological processes vital for maintaining genome integrity and should be central to the choice of PARPi-based chemotherapy and treatment strategies. Furthering our understanding of the PARylation metabolism, how covalent and non-covalent PARylation influence DDR protein dynamics and interactions, and how both contribute to the underlying mechanism of PARPi sensitivity and resistance is critical to improving their efficacy.

## Data Availability

No new data were generated or analysed in support of this research.
